# Use of medicines recommended for secondary prevention of acute coronary syndrome

**DOI:** 10.1590/S0034-8910.2015049005978

**Published:** 2015-12-16

**Authors:** Mari Ângela Gaedke, Juvenal Soares Dias da Costa, Euler Roberto Fernandes Manenti, Ruth Liane Henn, Vera Maria Vieira Paniz, Marcelo Felipe Nunes, Monique Adriane da Motta, Maria Teresa Anselmo Olinto

**Affiliations:** I Programa de Pós-Graduação em Saúde Coletiva. Universidade do Vale do Rio dos Sinos. São Leopoldo, RS, Brasil; II Departamento de Enfermagem e Odontologia. Universidade de Santa Cruz do Sul. Santa Cruz do Sul, RS, Brasil; III Instituto de Medicina Vascular do Hospital Mãe de Deus. Porto Alegre, RS, Brasil; IV Departamento de Nutrição. Universidade Federal de Ciências da Saúde de Porto Alegre. Porto Alegre, RS, Brasil

**Keywords:** Acute Coronary Syndrome, Secondary Prevention, Medication Adherence, Evidence-Based Medicine, Cohort Studies

## Abstract

**OBJECTIVE:**

: To analyze if the demographic and socioeconomic variables, as well as percutaneous coronary intervention are associated with the use of medicines for secondary prevention of acute coronary syndrome.

**METHODS:**

: In this cohort study, we included 138 patients with acute coronary syndrome, aged 30 years or more and of both sexes. The data were collected at the time of hospital discharge, and after six and twelve months. The outcome of the study was the simultaneous use of medicines recommended for secondary prevention of acute coronary syndrome: platelet antiaggregant, beta-blockers, statins and angiotensin-converting-enzyme inhibitor or angiotensin receptor blocker. The independent variables were: sex, age, education in years of attending, monthly income in tertiles and percutaneous coronary intervention. We described the prevalence of use of each group of medicines with their 95% confidence intervals, as well as the simultaneous use of the four medicines, in all analyzed periods. In the crude analysis, we verified the outcome with the independent variables for each period through the Chi-square test. The adjusted analysis was carried out using Poisson Regression.

**RESULTS:**

: More than a third of patients (36.2%; 95%CI 28.2;44.3) had the four medicines prescribed at the same time, at the moment of discharge. We did not observe any differences in the prevalence of use in comparison with the two follow-up periods. The most prescribed class of medicines during discharge was platelet antiaggregant (91.3%). In the crude analysis, the demographic and socioeconomic variables were not associated to the outcome in any of the three periods.

**CONCLUSIONS:**

: The prevalence of simultaneous use of medicines at discharge and in the follow-ups pointed to the under-utilization of this therapy in clinical practice. Intervention strategies are needed to improve the quality of care given to patients that extend beyond the hospital discharge, a critical point of transition in care.

## INTRODUCTION

Cardiovascular diseases are the leading cause of disability and morbidity and mortality worldwide, affecting developed and developing countries.^27^ Much of the burden of cardiovascular disease is due to acute coronary syndrome (ACS). This syndrome includes acute myocardial infarction (AMI) with and without ST segment elevation and unstable angina (UA), and is characterized by ischemia and necrosis of the heart muscle due to an atherothrombotic event.^11^

Ischemic events accumulate high complication rates after the acute phase. Therefore, recommendations for therapy based on clinical evidence have been developed. Consequently, recent advances in the treatment of ACS show decline in mortality and recurrence of events.^10,20^

As for secondary prevention of ACS, the continuous use and for an indefinite period of blood platelet antiaggregants, beta-blockers, statins and angiotensin-converting-enzyme inhibitors (ACE) is recommended for the secondary prevention of ACS. In case of intolerance to ACE, the use of angiotensin receptor blockers (ARB) is recommended. These conducts are supported consensually by national and international clinical practice guidelines in the area of cardiology.^13,19,20,22^ In addition, although these drugs individually may be effective for the reduction of morbidity and mortality after the ACS, its simultaneous and extended use present even better results,^15^ the prescription being made during critical discharge and also being decisive for these results.^2,12^ Additionally, the percutaneous coronary intervention with coronary stent implantation is one of the treatment options for the ACS, and it can be divided into: primary (without the use of blood thinners); facilitated (related to the use of previous pharmacology); and of rescue (due to failure of fibrinolysis), in this case, it is practiced electively after fibrinolysis.^20^

Among the four classes of medicines mentioned, blood platelet antiaggregants are the most prescribed, with recommended usage to all patients for an indefinite period. In patients who did percutaneous coronary intervention with stent placement, the use of antiplatelet double blockage has been recommended for at least 12 months with simultaneous use of acetylsalicylic acid (ASA) and addition of a P2Y inhibitor.^12,13,19,22^ A study pointed out reduction in the risk of cardiovascular death and AMI using double blockage.^17^ However, the therapy is characterized by its long duration, with regular and systematic follow-up, which hinders the attention to these patients.^21^

Low adhesion to the treatment of chronic diseases in the long term is a global problem. It causes medical and social complications with reduction in the quality of life and increased healthcare spending. The adhesion of patients is approximately 50.0% in developed countries and even lower in developing countries.^26^

Studies on the prevalence of the use of secondary prevention therapy in ACS are mainly carried out in developed countries. Therefore, data about the Brazilian reality are scarce. Studies point to the underutilization of therapy in clinical practice despite the large volume of evidence that sustain it.^9,25^

The availability of information on the use of these medicines in the population gives the opportunity to monitor the quality of care provided, identifying needs in in-hospital care and follow-up required after discharge, to decrease complications that may result in hospital readmissions and mortality.

Thus, this study aimed to analyze if demographic and socioeconomic variables, as well as percutaneous coronary interventions are associated with the use of medicines for secondary prevention of acute coronary syndrome.

## METHODS

In this cohort study, 138 patients were included, aged 30 years or more, of both sexes, with a diagnosis of acute coronary syndrome (unstable angina, AMI without ST segment elevation and AMI with ST segment elevation) from a large hospital in the city of Porto Alegre, RS, Southern Brazil. The hospital is a charity institution, with approximately 400 beds, and answers mostly private and insurance-covered patients. The data were collected at the time of hospital discharge, and after six and twelve months.

The exclusion criteria for patients were: residing outside of Rio Grande do Sul; not having a telephone number for contact; having their diagnosis changed over the period of hospitalization to another not included in criteria for inclusion; and being unable to respond to the questionnaire or not having family or someone responsible for them available in the hospital after three attempts of contact.

The sample size was estimated according to the following parameters: 95% confidence interval (95%CI); 80.0% statistical power; exposed and non-exposed ratio of 1:2, according to distribution of family income; outcome frequency in the non-exposed group of 30.0%; and relative risk of 2.0. Thus, the participation of 109 individuals would be necessary, plus 25.0% corresponding to loss and possibility of adjusted analysis, totaling 136 participants. Throughout the follow-ups, thirteen patients were classified as loss, refusal, or death at six months; and eight patients between six months and one year.

A pilot study was carried out during the first 30 days of research to assess the quality of data collection instruments and to check field logistics. Quality control was conducted on random sample of 5.0% of people included in the study to assess the internal validity of the survey.

Trained interviewers collected the data using three models of standardized and previously coded questionnaires. Demographic and socioeconomic data were extracted from a basal questionnaire applied directly to patients during hospitalization. Clinical information were supplemented using their medical records. Data referring to medical diagnostic according to the code of the International Classification of Diseases (ICD-10) and medicines used during hospital discharge were collected from the medical records in a retrospective query. Two questionnaires were applied through telephone contact in the follow-ups of six months and a year after hospital discharge. The information on the use of medicines were self-reported, including waiting for verification of medicines available in the homes of the participants.

The outcomes of the study were: simultaneous use of medicines recommended by scientific evidence for secondary prevention of ACS (considering any platelet antiaggregant, beta-blocker, ACE or ARB and statin,^13,22^ and use of antiplatelet double blockage (simultaneous use of ASA and a P2Y12 inhibitor).^19^ The outcomes were analyzed regarding exposure in three moments: discharge, follow-up of six months and follow-up of one year after discharge.

The independent variables were: sex (male; female), age (30 to 49 years; 50 to 64 years; and 65 or more), education in years of attending school (up to 11; 12 or more), family monthly income at tertiles (lower tertile – less than R$2,000.00; medium tertile R$2,000.00 to R$6,000.00; higher tertile – more than R$6,000.00), and percutaneous coronary intervention (no; yes).

Data entry was performed in the program EpiInfo by double typing, so that consistency between the two bases could be established and any discrepancy of values could be verified in the original questionnaires.

We described the prevalence of occurrence of the use of each group of medicine, as well as their simultaneous use in three moments, discharge, follow-up at six months and follow-up at one year. Prevalence estimates were compared by the respective 95%CI. The crude analysis characterized the population and estimated the prevalence of outcomes (simultaneous use of four classes of medicines and use of antiplatelet double blockage), and their association with independent variables for each period through the Chi-square test, with significance if p < 0.05. Statistical analysis was performed using the software SPSS 17 (Statistical Package for the Social Sciences).

The adjusted analysis was conducted in the Stata 11 Program, using Poisson regression with robust variance, and we obtained as effect measure the prevalence ratio. The variables whose statistical test result was less than 0.20 were inserted in the model, establishing significance if p ≤ 0.05.

The research project was approved by the Research Ethics Committee of the Universidade Vale do Rio dos Sinos (UNISINOS – Resolution 091/2008 of December 9, 2008).

## RESULTS

Little more than 1/3 (36.2%) of patients received prescriptions of the four classes of recommended medicines simultaneously during discharge. In comparison with the two following periods (six months to a year), the 95%CI overlapped, indicating absence of differences between the estimates of prevalence of use of the recommended medicines (Table 1).

**Table 1:** Prevalence of use of each class of medication and simultaneous use of four classes at hospital discharge, at six months and one year of follow-up. Rio Grande do Sul, Southern Brazil, 2013.

Medicine	Discharge (n = 138)		Six months (n = 125)		One year (n = 117)	

	%	95%CI	%	95%CI	%	95%CI
ACE/ARB	54.3	46.0; 62.7	52.0	43.2;60.8	56.4	47.4;65.4
Platelet antiaggregant	91.3	86.6;96.0	77.6	70.3;84.9	74.4	66.4;82.3
ASA	81.2	74.6;87.7	64.0	55.6;72.4	61.5	52.7;70.4
P2Y12 Inhibitor	74.6	67.4;81.9	44.0	35.3;52.7	40.2	31.3;49.1
Double blockage	64.5	56.5;72.5	30.4	22.3;38.5	27.4	19.3;35.4
Statin	86.2	80.5;92.0	76.8	69.4;84.2	79.5	72.2;86.8
Beta-blockers	70.3	62.7;77.9	68.0	59.8;76.2	70.1	61.8;78.4
Simultaneous use	36.2	28.2;44.3	36.0	27.6;44.4	34.2	25.6;42.8

ACE/ARB: angiotensin-converting-enzyme inhibitors/angiotensin receptor blocker; ASA: acetylsalicylic acid

The most prescribed class of medicines during discharge was platelet antiaggregant (91.3%), followed by statin (86.2%). The less prescribed class was ACE/ARB (54.3%) (Table 1).

The use of platelet antiaggregant decreased between discharge and the two follow-up periods. In the other classes of medications, we found no differences between the prevalence of use in all periods (Table 1).

Analyzing separately the rate of use of platelet antiaggregants regarding the use of ASA, P2Y12 inhibitors or antiplatelet double blockage, 81.2% of patients had isolated prescription of ASA and 74.6% had some 2Y12 inhibitor prescribed separately during discharge. Regarding double blockage, we observed a prevalence of 64.5% during discharge. The frequency of use decreased in every category when comparing with follow-up periods (Table 1).

Most patients were male (55.6%), with a average age of 68.0 years, average schooling of 13.6 years of attending school and average income around eight minimum wages (Table 2).

**Table 2:** Description of the sample and prevalence of simultaneous use of the four classes of medicines regarding the demographic and socioeconomic variables, as well as percutaneous coronary intervention in the three monitoring periods. Rio Grande do Sul, Southern Brazil, 2013.

Variable	n	%	Discharge		Six months		One year	

			Prevalence %	p	Prevalence %	p	Prevalence %	p
Sex				0.46^a^		0.17^a^		0.41^a^
Female	56	44.4	39.3		28.8		29.8	
Male	70	55.6	32.9		42.9		39.0	
Age (years)				0.36^b^		0.36^b^		0.80^b^
30 to 49	14	11.1	35.7		50.0		27.3	
50 to 64	38	30.2	44.7		35.0		37.5	
65 or more	74	58.7	31.1		34.3		34.9	
Schooling (in years of attending)				0.35^a^		1.0^a^		0.84^a^
12 or more	69	54.8	31.9		36.5		36.2	
Up to 11	57	45.2	40.4		36.5		33.3	
Income in tertile				0.50^b^		0.48^b^		0.80^b^
Higher tertile	42	33.3	40.5		31.6		32.4	
Medium tertile	42	33.3	33.3		38.5		36.8	
Lowest tertile	42	33.3	33.3		39.5		35.3	
Percutaneous coronary intervention				0.58^a^		0.48^a^		0.24^a^
No	69	54.8	33.3		36.9		33.0	
Yes	57	45.2	38.6		50.0		50.0	

^a^ Pearson’s Chi-square.

^b^ Linear trend test.

In the crude analysis, we examined the association of the simultaneous use of the four medicines with demographic and socioeconomic variables, as well as percutaneous coronary intervention. The results did not show any statistically significant differences in the three periods (Table 2).

The crude analysis of demographic and socioeconomic variables in relation to the use of antiplatelet double blockage showed higher prevalence among men compared to women in the follow-ups of six months and a year. We observed a higher prevalence of use of these drugs among people with higher education in the follow-ups of six months and a year. We also verified this outcome concerning percutaneous coronary intervention in all follow-ups (Table 3).

**Table 3:** Description of the sample and prevalence of use of double antiplatelet blockage regarding the demographic and socioeconomic variables, as well as percutaneous coronary intervention in the three monitoring periods. Rio Grande do Sul, Southern Brazil, 2013.

Variable	n	%	Discharge		Six months		One year	

			Prevalence %	p	Prevalence %	p	Prevalence %	p
Sex				0.27^a^		0.004^a^		0.007^a^
Female	56	44.4	58.9		15.4		12.8	
Male	70	55.6	68.6		41.3		37.3	
Age (years)				0.22^b^		0.44^b^		0.93^b^
30 to 49	14	11.1	71.4		35.7		18.2	
50 to 64	38	30.2	71.1		32.4		31.3	
65 or more	74	58.7	59.5		26.9		25.4	
Schooling (in years of attending)				0.1^a^		0.001^a^		0.047^a^
12 or more	69	54.8	71.0		42.9		34.5	
Up to 11	57	45.2	56.1		13.5		16.7	
Income in tertile				0.26^b^		0.13^b^		0.17^b^
Higher tertile	42	33.3	69.0		31.6		35.3	
Medium tertile	42	33.3	66.7		41.0		23.7	
Lowest tertile	42	33.3	57.1		15.8		20.6	
Percutaneous coronary intervention				0.003^a^		0.01^a^		0.06^a^
No	69	54.8	52.2		28.8		25.0	
Yes	57	45.2	78.9		75.0		50.0	

^a^ Pearson’s Chi-square.

^b^ Linear trend test.

Percutaneous coronary intervention remained associated with the use of antiplatelet double blockage in the adjusted analysis in relation to hospital discharge: those who performed it showed 50.0% higher prevalence of use antiplatelet double blockage (Table 4).

**Table 4:** Adjusted prevalence ratio and their respective 95% confidence intervals (95%CI) of the use of double antiplatelet blockage according to demographic and socioeconomic variables, as well as percutaneous coronary intervention in the three follow-up periods. Rio Grande do Sul, Southern Brazil, 2013.

Variable	Discharge			Six months			One year		

	Adjusted analysis			Adjusted analysis			Adjusted analysis		

	RP	95%CI	p	RP	95%CI	p	RP	95%CI	p
Sex						0.06^b^			0.03^b^
Female	^a^			1			1		
Male				1.9	0.98;3.90		2.4	1.08;5.54	
Age (years)			0.69^c^						
30 to 49	1								
50 to 64	1.0	0.73;1.53		^a^			^a^		
65 or more	0.96	0.65;1.42							
Schooling (in years of attending)			0.13^b^			0.02^b^			0.47^b^
12 or more	1			1			1		
Up to 11	0.8	0.60;1.06		0.4	1.88;0.85		0.8	0.38;1.57	
Income in tertile						0.84^c^			0.63^c^
Higher tertile	^a^			1			1		
Medium tertile				1.5	0.86;2.60		0.7	0.34;1.35	
Lowest tertile				0.9	0.40;2.26		0.9	0.39;1.97	
Percutaneous coronary intervention			0.002^b^			0.002^b^			0.09^b^
No	1			1			1		
Yes	1.5	1.16;1.96		2.0	1.30;3.21		1.8	0.91;3.43	

^a^ We included in the adjusted analysis only the variables with p < 0.2 in the crude analysis.

^b^ Wald Test for heterogeneity of proportions.

^c^ Wald Test for linear trend.

They remained associated with schooling and percutaneous coronary intervention in the follow-up of six months after adjustment. Those with higher education had 40.0% higher prevalence of use of antiplatelet double blockage, and those who undergone intervention showed twice as much use (Table 4).

In the one-year follow-up, only the variable sex was associated with the use of antiplatelet double blockage after adjustment. The prevalence of use of double blockage in men was twice more than in women. Percutaneous coronary intervention showed higher prevalence, but without statistical significance after adjustment (Table 4).

We analyzed the group of 50 patients who used the four classes at the moment of hospital discharge (36.2%) in two follow-ups. At six months, 18.4% was still using the four classes, which corresponded to a decrease of 49.2% in adhesion to the recommendations. At one-year follow-up, 17.1% of that group kept using the four classes (a decrease of 52.8% compared to the moment of discharge).

Among those who were not prescribed the four classes at discharge, 27.5% of individuals began to make simultaneous use of the medicines in the six months follow-up. Between six months and one year, 27.4% began to use the four medicines, resulting in similar proportions in the three moments (Table 1).

## DISCUSSION

The prevalence of simultaneous use of medicines at discharge and in follow-ups indicated underutilization in clinical practice, and has not been associated with demographic and socioeconomic variables, and neither percutaneous coronary intervention. Thus, it is necessary to invest in intervention strategies that improve the quality of attention paid to this group of patients.

ACS is an important public health problem. Therefore, it is necessary to search for secondary prevention interventions that aim at reducing complications and mortality. The clinical practice guidelines that follow evidence-based medicine recommend the use of the four groups of safe and effective medicines, individually^13,19,22^ or associated, to reduce mortality after ACS and recurring events.^15^ In addition, simultaneous use of these medicines indicates the quality of care provided.

The quality of care in health is measured by attributes such as access, equity, and effectiveness obtained at an endurable cost to society.^6^ In addition, the effectiveness of health care is determined by the diagnostic ability of the services and by the adhesion both of the person responsible for action (professionals) and the patient.^7^ Professional adhesion includes, among others, the adoption of safe procedures and prescription medicines of recognized effectiveness, according to the best evidence. Thus, this study may be considered as a marker of quality of the care provided, because the prescription at discharge measured compliance to the doctor’s recommendations. In turn, the maintenance of the treatment prescribed in the follow-ups reflected the patients’ adhesion. Both can be influenced by the presence of contraindications or emergence of adverse effects.

The prevalence of simultaneous use of any platelet antiaggregant, beta-blockers, ACE/ARB and statin found in this study (around 36.0%) at discharge and follow-ups indicated underutilization of this therapy in clinical practice, despite the large number of evidence that they held. Most studies on the subject showed this same situation. The prevalence of prescription of all four medicines was 35.6% in a cohort monitored in Canada with 5,833 patients with ACS.^24^ A cohort study^16^ with 1,135 patients analyzed the rates of use of three medicines based on evidence (ACE/ARB, beta-blockers and statins) in the Middle Atlantic States in the USA. Most patients with ACS after hospital discharge received at least one of the recommended classes. However, for 70.0% of these, at least one of the medicines were missing.^16^ An observational French study carried out with 1,700 patients showed that the four medicines were prescribed at discharge to 46.2% of the individuals.^2^ These prevalences were included in the confidence interval of the frequency found in this study (95%CI 28.2;44.3), showing no differences concerning the findings published in other countries.

A study carried out in Brazil^4^ assessed the effect of multi-faceted educational interventions for improving quality in prescribing evidence-based therapies. The results showed that the use of all therapies during the first 24h of hospitalization and at hospital discharge between the eligible patients was higher in the intervention group compared to the control (50.9% *versus* 31.9%; p = 0.03). However, the concomitant use of aspirin, beta-blockers, statins, and ACE at hospital discharge was 65.9% in the group that received interventions and 56.6% in the control group (p = 0.23).

In this study, we presented the prevalence to each medicine at discharge. The results were similar to other studies, such as in the observational French study, which found a rate of 82.4% for beta-blockers, 98.9% for platelet antiaggregants, 89.2% for statins, and 58.0% for ACE.^2^

The results of the Global Registry of Acute Coronary Events (GRACE)^9^ showed consistence and similarities regarding this study. About 90.0% of patients used platelet antiaggregants at hospital discharge; around 55.0%, ACE with small geographical differences; and 71.0%, beta-blockers. The prevalence of the use of statins was 47.0% in the GRACE, with geographical variations in its use; of 26.0% in Argentina and Brazil to 57.0% in Australia, New Zealand and Canada^9^ – below the 86.2% observed in this analysis.

The low prevalence for the use of ACE/ARB influenced the prevalence of simultaneous use of the four medicines. The use of this class of medicines was a class II recommendation, with A level of evidence,^19,22^ unlike other classes of medicines that were class I recommendation and A level of evidence for treating ACS.

The simultaneous use of the four medicines reached low prevalence. We analyzed each medicine separately and the use of platelet antiaggregants obtained best incorporation to clinical practice, since 91.3% of patients received at discharge at least one antiaggregant. This data confirmed what the best evidence have shown. ASA is considered the best antiplatelet, and it is a consensus that its use in secondary prevention of ACS, indefinitely, regardless of its clinical form.^13,19^ The current guidelines show that therapy with platelet P2Y12 receptor inhibitors should be added to the use of ASA, during 12 months, particularly for those patients who performed percutaneous coronary intervention, with metallic or pharmacological stent placement.^13,19,22^

The data collection instrument did not measure whether the low prevalence in the follow-up was associated with non-adhesion of patient to treatment or non-prescription of the medicines by the medical professional. Neither the establishment of the involvement of other issues such as access to the medicine or emergence of side effects was allowed. The sample size may have prevented the meeting of the surveyed associations.

The population of this study, from a single hospital, presented high income and schooling when compared to those of the Brazilian population, in addition to being users of health insurances or privately covered by the health service. In addition, although the analysis has not shown any significant association regarding age, the study population was predominantly of older adults. The low prevalence found can be partly explained by the possible occurrence of clinical contraindications to the use of these medicines, or emergence of adverse effects, but hardly explained by difficult access due to financial reasons.

No difference was observed in the prevalence of use of each medicine separately throughout follow-ups, except for platelet antiaggregants. An important decrease of use occurred between discharge and follow-up periods regarding the use of ASA, P2Y12 receptor inhibitor or double blockage, contrary to the recommendations advocated by the guidelines. This decline may have been caused by concerns about the risk-benefit of these medications, especially by increasing the risk of bleeding, since the population was predominantly of older adults and due to the cost of P2Y12 receptor inhibitors, as the literature points out.^1,5^ Other studies consistently designed and well conducted showed decrease in prevalence of use of antiplatelet double blockage at the one year follow-up.^8,23^

The prevalence of the use of antiplatelet double blockage decreased between the follow-ups. However, the antiplatelet dual blockage was better incorporated by those individuals who performed percutaneous coronary intervention in almost all periods. Individuals who have been affected by the intervention presented 77.0% higher prevalence of use at the one year follow-up. However, not having done any intervention was associated with the outcome, perhaps a reflection of the power of the study due to the size of the sample. We noticed the existence of a critical point for the non-adherence to recommended treatment between the follow-ups of six months and one year.

About 50.0% of patients kept the simultaneous use of the four medicines during the follow-up periods, resulting in low adhesion to the treatment recommended by the guidelines. Other studies indicate the same proportion of adhesion to long-term treatments, with results varying from 45.6% to 54.0%.^2,14^ A study with 1,077 patients with ACS showed that 1/3 of them have ceased to use at least one of the recommended medications prescribed within three months of discharge.^18^

In the follow-ups, we investigated who began to use the medicines simultaneously. About 30.0% of patients began to use the therapy recommended by guidelines in the two periods examined. A similar result was found by a multicenter study in Canada, in which 77.0% of patients that did not receive proper treatment in the hospital remained without proper treatment after one year.^3^

Despite of the lack of national studies for comparison of results, the prevalences found are close to studies conducted in other countries.

The study presented obstacles that seem to exist between the evidence and the effectiveness of these treatments in clinical practice. It exposed the need of developing intervention strategies to improve the quality of care given to patients that extend beyond the hospital discharge, a critical point of transition in care.
