# Supplementary material for: Use of medicines recommended for secondary prevention of acute coronary syndrome
Source: Rev Saude Publica. 2015 Dec 16;49:88. doi: 10.1590/S0034-8910.2015049005978 (PMC4687825; doi:10.1590/S0034-8910.2015049005978)
Supplement: Translation

# Translation: Uso de medicamentos recomendados na prevenção secundária da síndrome coronariana aguda

**Authors:** Mari Ângela Gaedke, Juvenal Soares Dias da Costa, Euler Roberto Fernandes Manenti, Ruth Liane Henn, Vera Maria Vieira Paniz, Marcelo Felipe Nunes, Monique Adriane da Motta, Maria Teresa Anselmo Olinto

**Affiliations:** I Programa de Pós-Graduação em Saúde Coletiva. Universidade do Vale do Rio dos Sinos. São Leopoldo, RS, Brasil; II Departamento de Enfermagem e Odontologia. Universidade de Santa Cruz do Sul. Santa Cruz do Sul, RS, Brasil; III Instituto de Medicina Vascular do Hospital Mãe de Deus. Porto Alegre, RS, Brasil; IV Departamento de Nutrição. Universidade Federal de Ciências da Saúde de Porto Alegre. Porto Alegre, RS, Brasil

**Keywords:** Síndrome Coronariana Aguda, Prevenção Secundária, Adesão à Medicação, Medicina Baseada em Evidências, Estudos de Coortes

## Abstract

**OBJETIVO:**

: Analisar se variáveis demográficas e socioeconômicas e intervenção coronária percutânea estão associadas ao uso de medicamentos para prevenção secundária da síndrome coronariana aguda.

**MÉTODOS:**

: Neste estudo de coorte, foram incluídos 138 pacientes com síndrome coronariana aguda, com idade igual ou superior a 30 anos e de ambos os sexos. Os dados foram coletados no momento da alta hospitalar e após seis e 12 meses da alta. O desfecho do estudo foi o uso simultâneo dos medicamentos recomendados para prevenção secundária da síndrome coronariana aguda: antiagregante plaquetário, betabloqueador, estatina e inibidor da enzima conversora de angiotensina ou bloqueador do receptor de angiotensina. As variáveis independentes foram: sexo, idade, escolaridade em anos de estudo, renda mensal em tercis e intervenção coronária percutânea. Descreveu-se a prevalência do uso de cada grupo de medicamento com respectivos intervalos de confiança de 95%, bem como o uso simultâneo dos quatro, em todos os períodos analisados. Na análise bruta verificou-se a associação do desfecho com as variáveis independentes para cada período por meio do teste Qui-quadrado. A análise ajustada foi realizada por Regressão de Poisson.

**RESULTADOS:**

: Mais de um terço dos pacientes (36,2%; IC95% 28,2;44,3) recebeu prescrição dos quatro medicamentos, simultaneamente, no momento da alta. Não foram observadas diferenças nas prevalências de uso na comparação com os dois períodos de seguimento. A classe de medicamento mais prescrita na alta foi antiagregante plaquetário (91,3%). Na análise bruta, as variáveis demográficas e socioeconômicas não se associaram ao desfecho em nenhum dos três períodos.

**CONCLUSÕES:**

: A prevalência de uso simultâneo dos medicamentos na alta e nos seguimentos apontou para a subutilização dessa terapêutica na prática clínica. São necessárias estratégias de intervenção para melhoria da qualidade da atenção dada aos pacientes que se estendam além da alta hospitalar, um ponto crítico de transição no cuidado.

## INTRODUÇÃO

As doenças cardiovasculares são a principal causa de incapacidade e morbimortalidade no mundo, atingindo países desenvolvidos e em desenvolvimento.^27^ Grande parte da carga das doenças cardiovasculares deve-se à síndrome coronariana aguda (SCA). Essa síndrome inclui o infarto agudo do miocárdio (IAM) com e sem elevação do segmento ST e a angina instável (AI), e se caracteriza pela isquemia e necrose do músculo cardíaco em decorrência de evento aterotrombótico.^11^

Os eventos isquêmicos acumulam taxas de complicações elevadas após a fase aguda. Por isso, têm sido desenvolvidas recomendações para a terapêutica com base em evidências clínicas. Consequentemente, os recentes avanços no tratamento da SCA mostram declínio na mortalidade e recorrência de eventos.^10,20^

Quanto à prevenção secundária da SCA, o uso contínuo e por tempo indeterminado de antiagregantes plaquetários, betabloqueadores, estatinas e inibidores da enzima conversora da angiotensina (IECA) é recomendado para a prevenção secundária da SCA. Em caso de intolerância aos IECA, preconizou-se o uso de bloqueador dos receptores de angiotensina (BRA). Essas condutas são apoiadas consensualmente por diretrizes clínicas nacionais e internacionais na área de cardiologia.^13,19,20,22^Além disso, apesar de esses medicamentos individualmente se mostrarem eficazes para a redução da morbimortalidade após a SCA, seu uso simultâneo e prolongado apresenta resultados ainda melhores,^15^ sendo a prescrição na alta hospitalar crítica e decisiva para esses resultados.^2,12^ Adicionalmente, a intervenção coronária percutânea com implantação de *stent* coronário é uma das opções de tratamento para a SCA, podendo ser dividida em: primária (sem o uso prévio de fibrinolíticos); facilitada (relacionada à utilização de farmacologia prévia); e de salvamento ou resgate (decorrente de insucesso da fibrinólise), neste caso, praticada de maneira eletiva após a fibrinólise.^20^

Dentre as quatro classes de medicamentos citadas, os antiagregantes plaquetários são os mais prescritos, com uso recomendado a todos os pacientes e por tempo indeterminado. Nos pacientes que fizeram intervenção coronária percutânea com colocação de *stent,* tem sido indicado bloqueio antiplaquetário duplo por, no mínimo, 12 meses com uso de ácido acetilsalicílico (AAS) e adição de um inibidor P2Y12.^13,19,22^ Estudo apontou redução no risco de morte cardiovascular e IAM com o uso do bloqueio duplo.^17^ Porém, a terapia caracteriza-se pela longa duração, com seguimento regular e sistemático, o que dificulta a atenção a esses pacientes.^21^

A baixa adesão ao tratamento de doenças crônicas em longo prazo é um problema mundial. Provoca complicações médicas e sociais com redução na qualidade de vida e aumento dos gastos dos sistemas de saúde. A adesão dos pacientes é de aproximadamente 50,0% em países desenvolvidos e ainda menor nos países em desenvolvimento.^26^

Estudos sobre a prevalência do uso da terapia de prevenção secundária na SCA são sobretudo realizados em países desenvolvidos, sendo escassos os dados acerca da realidade brasileira. Estudos apontam para a subtilização da terapêutica na prática clínica apesar do grande volume de evidências que a sustentam.^9,25^

A disponibilidade de informações sobre o uso desses medicamentos na população oferece a oportunidade de monitorar a qualidade do cuidado prestado, identificando necessidades na prestação do cuidado intra-hospitalar e no acompanhamento necessário no pós-alta, a fim de diminuir complicações que resultem em readmissões hospitalares e na mortalidade.

Assim, este estudo teve por objetivo analisar se variáveis demográficas e socioeconômicas e intervenção coronária percutânea estão associadas ao uso de medicamentos para prevenção secundária da síndrome coronariana aguda.

## MÉTODOS

Neste estudo de coorte, foram incluídos 138 pacientes de 30 anos ou mais, de ambos os sexos, com diagnóstico de síndrome coronariana aguda (angina instável, IAM sem supradesnivelamento do segmento ST e IAM com supradesnivelamento do segmento ST) oriundos de hospital de grande porte no município de Porto Alegre, RS. Trata-se de entidade filantrópica, com aproximadamente 400 leitos, e que presta principalmente atendimentos privados e a convênios. Os dados foram coletados no momento da alta hospitalar e aos seis e 12 meses após a alta.

Foram excluídos os pacientes: que residiam fora do Rio Grande do Sul; que não possuíam telefone para serem contatados; que tiveram seu diagnóstico alterado ao longo do período de hospitalização para outro não contemplado nos critérios de inclusão; e que estavam incapacitados de responder ao questionário ou que não possuíam familiar ou responsável acessível no hospital após três tentativas de contato.

O tamanho da amostra foi estimado segundo os seguintes parâmetros: intervalo de 95% de confiança (IC95%); 80,0% de poder estatístico; razão de expostos e não expostos de 1:2, conforme distribuição de renda familiar; frequência do desfecho no grupo não exposto de 30,0%; e risco relativo de 2,0. Assim, seria necessário o ingresso de 109 indivíduos, acrescido de 25,0% correspondente às perdas e possibilidade de análise ajustada, totalizando 136 participantes. Ao longo dos seguimentos, treze pacientes foram classificados como perda, recusa ou óbito aos seis meses, e oito pacientes entre seis meses e um ano.

Foi realizado estudo piloto durante os primeiros 30 dias de pesquisa para avaliar a qualidade dos instrumentos de coleta de dados e verificar a logística do trabalho de campo. O controle de qualidade foi conduzido em amostra aleatória de 5,0% das pessoas incluídas no estudo para avaliar a validade interna da pesquisa.

Entrevistadores treinados coletaram os dados utilizando três modelos de questionários padronizados e pré-codificados. Os dados demográficos e socioeconômicos foram extraídos de questionário basal aplicado diretamente aos pacientes durante a internação hospitalar, informações clínicas foram complementadas usando-se os respectivos prontuários médicos. Os dados referentes ao diagnóstico médico conforme código da Classificação Internacional das Doenças (CID-10) e medicamentos usados na alta hospitalar foram coletados de prontuários em consulta retrospectiva. Dois questionários foram aplicados por meio de contato telefônico nos acompanhamentos de seis meses e um ano após alta hospitalar. As informações sobre o uso de medicamentos foram autorrelatadas, incluindo espera para verificação dos medicamentos disponíveis nas residências dos participantes.

Os desfechos do estudo foram: uso simultâneo dos medicamentos recomendados pelas evidências científicas para prevenção secundária da SCA (considerando qualquer antiagregante plaquetário, betabloqueador, IECA ou BRA e estatina,^13,22^ e uso de bloqueio antiplaquetário duplo (uso simultâneo de AAS e um inibidor P2Y12).^19^ Os desfechos foram analisados em relação às exposições em três momentos: alta hospitalar, seguimento de seis meses e de um ano após a alta.

As variáveis independentes foram: sexo (masculino; feminino), idade (30 a 49 anos; 50 a 64 anos; e 65 ou mais), escolaridade em anos de estudo (até 11; 12 ou mais), renda mensal familiar em tercis (tercil mais baixo – menos de R$2.000,00; tercil intermediário – de R$2.000,00 a R$6.000,00; tercil mais alto – mais de R$6.000,00) e intervenção coronária percutânea (não; sim).

A entrada dos dados foi realizada no programa EpiInfo por dupla digitação, a fim de que a consistência entre os dois bancos pudesse ser estabelecida e qualquer discrepância de valores fosse conferida nos questionários originais.

Descreveu-se a prevalência de ocorrência do uso de cada grupo de medicamento, bem como o uso simultâneo das quatro classes nos três momentos, alta hospitalar, seguimento aos seis meses e um ano. As estimativas das prevalências foram comparadas pelos respectivos IC95%. A análise bruta caracterizou a população e estimou a prevalência dos desfechos (uso simultâneo das quatro classes de medicamentos e uso de bloqueio antiplaquetário duplo) e sua associação com as variáveis independentes para cada período por meio do teste Qui-quadrado, com significância se p < 0,05. A análise estatística foi realizada no programa SPSS 17 (*Statistical Package for the Social Sciences*).

A análise ajustada foi realizada no programa Stata versão 11, por meio de Regressão de Poisson com variância robusta, obtendo-se como medida de efeito a razão de prevalência. Ingressaram no modelo as variáveis cujo resultado do teste estatístico foi menor que 0,20, estabelecendo-se significância se p ≤ 0,05.

O projeto de pesquisa foi aprovado pelo Comitê de Ética em Pesquisa da Universidade do Vale do Rio dos Sinos (UNISINOS – Resolução 091/2008 de 9 de dezembro de 2008).

## RESULTADOS

Pouco mais de 1/3 (36,2%) dos pacientes receberam prescrição na alta simultaneamente das quatro classes de medicamentos preconizadas. Na comparação com os dois períodos de seguimento (seis meses e um ano), os IC95% se sobrepuseram, indicando que não havia diferenças entre as estimativas de prevalências de uso dos medicamentos recomendados (Tabela 1).

**Tabela 1:** Prevalência de uso de cada classe de medicamento e do uso simultâneo das quatro classes na alta hospitalar, aos seis meses e ao um ano de acompanhamento. Rio Grande do Sul, 2013.

Medicamento	Alta (n = 138)		Seis meses (n = 125)		Um ano (n = 117)	

	%	IC95%	%	IC95%	%	IC95%
IECA/BRA	54,3	46,0;62,7	52,0	43,2;60,8	56,4	47,4;65,4
Antiagregante plaquetário	91,3	86,6;96,0	77,6	70,3;84,9	74,4	66,4;82,3
AAS	81,2	74,6;87,7	64,0	55,6;72,4	61,5	52,7;70,4
Inibidor P2Y12	74,6	67,4;81,9	44,0	35,3;52,7	40,2	31,3;49,1
Bloqueio duplo	64,5	56,5;72,5	30,4	22,3;38,5	27,4	19,3;35,4
Estatina	86,2	80,5;92,0	76,8	69,4;84,2	79,5	72,2;86,8
Betabloqueador	70,3	62,7;77,9	68,0	59,8;76,2	70,1	61,8;78,4
Uso simultâneo	36,2	28,2;44,3	36,0	27,6;44,4	34,2	25,6;42,8

IECA/BRA: inibidores da enzima conversora de angiotensina/bloqueador do receptor de angiotensina; AAS: ácido acetilsalicílico

A classe de medicamentos mais prescrita na alta foi de antiagregante plaquetário (91,3%), acompanhada pela estatina (86,2%). A classe menos prescrita na alta foi IECA/BRA (54,3%) (Tabela 1).

O uso de antiagregante plaquetário diminuiu entre a alta e os dois períodos de seguimento. Nas demais classes de medicamentos, não foram observadas diferenças entre as prevalências de uso em todos os períodos (Tabela 1).

Ao se analisar separadamente a prevalência de uso dos antiagregantes plaquetários quanto ao uso de AAS, inibidores P2Y12 ou ao bloqueio antiplaquetário duplo, 81,2% dos pacientes tiveram prescrição isolada de AAS e 74,6% tiveram algum inibidor P2Y12 prescrito isoladamente na alta. Em relação ao bloqueio duplo, observou-se prevalência de 64,5% na alta. A frequência de uso diminuiu em todas as categorias ao se comparar com os períodos de seguimento (Tabela 1).

A maioria dos pacientes era do sexo masculino (55,6%), com mediana de idade de 68,0 anos, média de escolaridade de 13,6 anos de estudo e renda média em torno de oito salários mínimos (Tabela 2).

**Tabela 2:** Descrição da amostra e prevalência de uso simultâneo das quatro classes de medicamentos em relação às variáveis demográficas, socioeconômicas e intervenção coronária percutânea nos três períodos de acompanhamento. Rio Grande do Sul, 2013.

Variável	n	%	Alta		Seis meses		Um ano	

			Prevalência %	p	Prevalência %	p	Prevalência %	p
Sexo				0,46^a^		0,17^a^		0,41^a^
Feminino	56	44,4	39,3		28,8		29,8	
Masculino	70	55,6	32,9		42,9		39,0	
Idade (anos)				0,36^b^		0,36^b^		0,80^b^
30 a 49	14	11,1	35,7		50,0		27,3	
50 a 64	38	30,2	44,7		35,0		37,5	
65 ou mais	74	58,7	31,1		34,3		34,9	
Escolaridade (em anos de estudo)				0,35^a^		1,0^a^		0,84^a^
12 ou mais	69	54,8	31,9		36,5		36,2	
Até 11	57	45,2	40,4		36,5		33,3	
Renda em tercis				0,50^b^		0,48^b^		0,80^b^
Tercil mais alto	42	33,3	40,5		31,6		32,4	
Tercil médio	42	33,3	33,3		38,5		36,8	
Tercil mais baixo	42	33,3	33,3		39,5		35,3	
Intervenção coronária percutânea				0,58^a^		0,48^a^		0,24^a^
Não	69	54,8	33,3		36,9		33,0	
Sim	57	45,2	38,6		50,0		50,0	

^a^ Teste do Qui-quadrado de Pearson.

^b ^Teste de tendência linear.

Na análise bruta, analisou-se a associação do uso simultâneo dos quatro medicamentos com as variáveis demográficas, socioeconômicas e intervenção coronária percutânea. Os resultados não mostraram diferenças estatisticamente significativas nos três períodos (Tabela 2).

A análise bruta das variáveis demográficas e socioeconômicas em relação ao uso de bloqueio antiplaquetário duplo mostrou maior prevalência entre os homens comparados às mulheres nos seguimentos de seis meses e um ano. Observou-se maior prevalência de uso desses medicamentos entre as pessoas de maior escolaridade nos seguimentos de seis meses e um ano. Verificou-se também associação deste desfecho em relação à intervenção coronária percutânea em todos os seguimentos (Tabela 3).

**Tabela 3:** Descrição da amostra e prevalência de uso de bloqueio antiplaquetário duplo em relação às variáveis demográficas, socioeconômicas e intervenção coronária percutânea nos três períodos de acompanhamento. Rio Grande do Sul, 2013.

Variável	n	%	Alta		Seis meses		Um ano	

			Prevalência %	p	Prevalência %	p	Prevalência %	p
Sexo				0,27^a^		0,004^a^		0,007^a^
Feminino	56	44,4	58,9		15,4		12,8	
Masculino	70	55,6	68,6		41,3		37,3	
Idade (anos)				0,22^b^		0,44^b^		0,93^b^
30 a 49	14	11,1	71,4		35,7		18,2	
50 a 64	38	30,2	71,1		32,4		31,3	
65 ou mais	74	58,7	59,5		26,9		25,4	
Escolaridade (em anos de estudo)				0,1^a^		0,001^a^		0,047^a^
12 ou mais	69	54,8	71,0		42,9		34,5	
Até 11	57	45,2	56,1		13,5		16,7	
Renda em tercis				0,26^b^		0,13^b^		0,17^b^
Tercil mais alto	42	33,3	69,0		31,6		35,3	
Tercil médio	42	33,3	66,7		41,0		23,7	
Tercil mais baixo	42	33,3	57,1		15,8		20,6	
Intervenção coronária percutânea				0,003^a^		0,01^a^		0,06^a^
Não	69	54,8	52,2		28,8		25,0	
Sim	57	45,2	78,9		75,0		50,0	

^a^ Teste do Qui-quadrado de Pearson.

^b^ Teste de tendência linear.

A intervenção coronária percutânea manteve-se associada ao uso de bloqueio antiplaquetário duplo na análise ajustada em relação à alta hospitalar: aqueles que a realizaram apresentaram 50,0% maior prevalência de uso antiplaquetário duplo (Tabela 4).

**Tabela 4:** Razão de prevalência ajustada e seus respectivos intervalos de confiança de 95% (IC95%) do uso de bloqueio antiplaquetário duplo de acordo com variáveis demográficas, socioeconômicas e intervenção coronária percutânea nos três períodos de seguimento. Rio Grande do Sul, 2013.

Variável	Alta			Seis meses			Um ano		

	Análise ajustada			Análise ajustada			Análise ajustada		

	RP	IC95%	p	RP	IC95%	p	RP	IC95%	p
Sexo						0,06^b^			0,03^b^
Feminino	^a^			1			1		
Masculino				1,9	0,98;3,90		2,4	1,08;5,54	
Idade (anos)			0,69^c^						
30 a 49	1								
50 a 64	1,0	0,73;1,53		^a^			^a^		
65 ou mais	0,96	0,65;1,42							
Escolaridade (em anos de estudo)			0,13^b^			0,02^b^			0,47^b^
12 ou mais	1			1			1		
Até 11	0,8	0,60;1,06		0,4	1,88;0,85		0,8	0,38;1,57	
Renda em tercis						0,84^c^			0,63^c^
Tercil mais alto	^a^			1			1		
Tercil médio				1,5	0,86;2,60		0,7	0,34;1,35	
Tercil mais baixo				0,9	0,40;2,26		0,9	0,39;1,97	
Intervenção coronária percutânea			0,002^b^			0,002^b^			0,09^b^
Não	1			1			1		
Sim	1,5	1,16;1,96		2,0	1,30;3,21		1,8	0,91;3,43	

^a^ Incluídas na análise ajustada apenas as variáveis com p < 0,2 na análise bruta.

^b^ Teste Wald para heterogeneidade de proporções.

^c^ Teste Wald para tendência linear.

Mantiveram-se associadas a escolaridade e intervenção coronária percutânea no seguimento de seis meses após o ajuste. Aqueles com escolaridade mais elevada tiveram 40,0% maior prevalência de uso de bloqueio antiplaquetário duplo, e os que sofreram a intervenção apresentaram duas vezes mais uso (Tabela 4).

Em um ano de seguimento, apenas a variável sexo manteve associação com o uso de bloqueio antiplaquetário duplo após o ajuste. Os homens apresentaram prevalência de uso do bloqueio duplo maior que duas vezes em relação às mulheres. A intervenção coronária percutânea mostrou maior prevalência, porém sem significância estatística após o ajuste (Tabela 4).

Foi realizada análise do grupo de 50 pacientes que usava as quatro classes na alta hospitalar (36,2%) em dois seguimentos. Aos seis meses, 18,4% ainda usava as quatro classes, o que correspondeu à diminuição de 49,2% na adesão às recomendações. No acompanhamento de um ano, 17,1% desse grupo permaneceram utilizando as quatro classes (diminuição de 52,8% em relação à alta hospitalar).

Entre aqueles que não tiveram as quatro classes prescritas na alta, 27,5% passaram a fazer o uso simultâneo dos medicamentos no seguimento de seis meses. Entre seis meses e um ano, 27,4% passaram a utilizar os quatro medicamentos, o que resultou em proporções semelhantes nos três momentos (Tabela 1).

## DISCUSSÃO

A prevalência do uso simultâneo dos medicamentos na alta e nos seguimentos indicou subutilização na prática clínica e não esteve associada a variáveis demográficas, socioeconômicas e intervenção coronária percutânea. Assim, é necessário investir em estratégias de intervenção que melhorem a qualidade da atenção dada a este grupo de pacientes.

A SCA representa importante problema de saúde pública. Isso implica a busca por intervenções de prevenção secundária que visem à redução de suas complicações e mortalidade. As diretrizes clínicas pautadas na medicina baseada em evidências recomendam o uso dos quatro grupos de medicamentos seguros e eficazes, individualmente^13,19,22^ ou em associação, na redução da mortalidade após SCA e em eventos recorrentes.^15^ Além disso, o uso simultâneo desses medicamentos é indicador da qualidade do cuidado prestado.

A qualidade dos cuidados em saúde é medida por atributos como acesso, equidade e efetividade obtidos a custo suportável para a sociedade.^6^ Além disso, a efetividade dos cuidados de saúde é determinada pela capacidade diagnóstica dos serviços e pela aderência tanto do responsável pela ação (profissionais) quanto do paciente.^7^ A aderência profissional inclui, entre outros aspectos, a adoção de procedimentos seguros e a prescrição de medicamentos de reconhecida eficácia, seguindo as melhores evidências. Desta forma, o presente estudo pode ser considerado como marcador da qualidade do cuidado prestado, pois a prescrição na alta mediu o seguimento médico às recomendações. Por sua vez, a manutenção do tratamento prescrito nos acompanhamentos nos seis meses e de um ano refletiu a adesão dos pacientes. Ambos podem ser influenciados pela presença de contraindicações ou surgimento de efeitos adversos.

A prevalência de uso simultâneo de qualquer antiagregante plaquetário, betabloqueador, IECA/BRA e estatina encontrada neste estudo (em torno de 36,0%) na alta e nos seguimentos indicou subutilização dessa terapêutica na prática clínica, apesar do grande número de evidências que as sustentavam. A maioria dos estudos acerca do tema mostrou esta mesma situação. A prevalência de prescrição na alta de todos os quatro medicamentos foi de 35,6% numa coorte acompanhada no Canadá com 5.833 pacientes com SCA.^24^ Estudo de coorte^16^ com 1.135 pacientes analisou as taxas de uso de três medicamentos baseados em evidências (IECA/BRA, betabloqueadores e estatinas) em estados do meio atlântico nos EUA. A maioria dos pacientes com SCA após a alta hospitalar recebia pelo menos uma das classes recomendadas. No entanto, para 70,0% destes faltava pelo menos um dos medicamentos.^16^ Estudo observacional francês com 1.700 pacientes mostrou que as quatro medicações foram prescritas na alta hospitalar para 46,2% dos indivíduos.^2^ Essas prevalências estavam incluídas no intervalo de confiança da frequência encontrada no presente estudo (IC95% 28,2;44,3), não apresentando diferenças em relação aos achados publicados em outros países.

Estudo realizado no Brasil^4^ avaliou o efeito de intervenções educativas multifacetadas para melhoria da qualidade na prescrição de terapias baseadas em evidências. Os resultados mostraram que o uso de todas as terapias durante as primeiras 24h de internação e na alta hospitalar entre os pacientes elegíveis foi maior no grupo intervenção em relação ao controle (50,9% *versus* 31,9%; p = 0,03). Entretanto, o uso concomitante de aspirina, betabloqueador, estatina e IECA na alta hospitalar foi de 65,9% no grupo que recebeu as intervenções e de 56,6% no controle (p = 0,23).

No presente estudo, apresentaram-se as prevalências para cada medicamento na alta. Os resultados foram semelhantes a outros estudos, como no estudo observacional francês, em que foi encontrada uma proporção de 82,4% para betabloqueadores, 98,9% para antiagregantes plaquetários, 89,2% para estatinas e 58,0% para IECA.^2^

Os resultados do Registro Global de Eventos Coronarianos Agudos (GRACE)^9^ indicaram consistência e semelhanças em relação ao presente estudo. Cerca de 90,0% dos pacientes usavam antiagregante plaquetário na alta hospitalar; em torno de 55,0%, IECA com pequenas diferenças geográficas; e 71,0%, betabloqueadores. A prevalência do uso de estatinas foi 47,0% no GRACE com variações geográficas no seu uso, de 26,0% na Argentina e Brasil a 57,0% na Austrália, Nova Zelândia e Canadá,^9^ portanto, inferior aos 86,2% constatados na presente análise.

A baixa prevalência para o uso de IECA/BRA influenciou na prevalência do uso simultâneo dos quatro medicamentos. O uso dessa classe de medicamento foi recomendação classe II e nível de evidência A,^19,22^ diferentemente das outras classes de medicamentos que possuíam recomendação classe I e nível de evidência A no tratamento da SCA.

O uso simultâneo dos quatro medicamentos alcançou baixa prevalência. Analisou-se separadamente cada medicamento e o uso de antiagregantes plaquetários obteve melhor incorporação à prática clínica, uma vez que 91,3% dos pacientes receberam na alta pelo menos um antiagregante. Este dado confirmou o que preconizaram as melhores evidências. O AAS é considerado o antiplaquetário de excelência e é consenso o seu uso na prevenção secundária da SCA, por tempo indeterminado, independentemente de sua forma clínica.^13,19^ As atuais diretrizes mostram que a terapia com inibidores do receptor P2Y12 plaquetário deveria ser adicionado ao uso do AAS, durante 12 meses, particularmente para aqueles pacientes que realizaram intervenção coronária percutânea, com colocação de *stent* metálico ou farmacológico.^13,19,22^

O instrumento de coleta de dados não permitiu medir se a baixa prevalência no seguimento estava associada a não adesão do paciente ao tratamento ou a não prescrição do medicamento pelo profissional médico. Tampouco permitiu estabelecer o envolvimento de outras questões como acesso ao medicamento ou surgimento de efeitos colaterais. O tamanho da amostra pode ter impossibilitado o encontro das associações pesquisadas.

A população deste estudo, proveniente de um único hospital, apresentou renda e escolaridade elevadas quando comparadas às da população brasileira, além de serem usuários de plano de saúde ou arcarem de forma privada pelo serviço de saúde. Além disso, embora a análise não tenha apresentado associação significativa em relação à idade, a população estudada foi predominantemente idosa. A baixa prevalência encontrada pode ser, em parte, explicada pela possível ocorrência de contraindicações clínicas ao uso desses medicamentos ou surgimento de efeitos adversos, mas dificilmente explicada por dificuldade de acesso por questões financeiras.

Não foi observada diferença nas prevalências de uso de cada medicamento, separadamente ao longo do seguimento, exceto para os antiagregantes plaquetários. Houve diminuição importante de uso entre a alta e os períodos de seguimento em relação ao uso de AAS, inibidor de receptor P2Y12 ou bloqueio duplo, contrariando as recomendações preconizadas pelas diretrizes. Essa diminuição pode ter sido causada por preocupações sobre o risco-benefício dessas medicações, principalmente pelo aumento do risco de sangramento, pela população ser predominantemente idosa e pelo custo dos inibidores dos receptores P2Y12, conforme a literatura aponta.^1,5^ Outros estudos com delineamento consistente e bem conduzidos mostraram diminuição da prevalência de uso de bloqueio antiplaquetário duplo no seguimento de um ano.^8,23^

A prevalência do uso de bloqueio antiplaquetário duplo diminuiu ao longo dos seguimentos. Porém, o bloqueio antiplaquetário duplo foi mais bem incorporado naqueles indivíduos que realizaram intervenção coronária percutânea em quase todos os períodos. Os indivíduos que sofreram a intervenção apresentaram 77,0% maior prevalência de uso no seguimento de um ano. Contudo, não ter realizado intervenção não se associou com o desfecho, talvez reflexo do poder do estudo devido ao tamanho da amostra. Pareceu haver um ponto crítico para a não adesão ao tratamento recomendado entre os seguimentos de seis meses e um ano.

Cerca de 50,0% dos pacientes mantiveram o uso simultâneo dos quatro medicamentos durante os períodos de seguimento, resultando em baixa adesão ao tratamento recomendado pelas diretrizes. Outros estudos apontam essa mesma proporção de adesão em tratamentos de longo prazo, com resultados variando entre 45,6% e 54,0%.^2,14^ Estudo com 1.077 pacientes com SCA mostrou que 1/3 deles deixaram de usar pelo menos um dos medicamentos preconizados prescritos dentro de três meses da alta hospitalar.^18^

Nos acompanhamentos, investigou-se quem passou a usar simultaneamente os medicamentos. Cerca de 30,0% dos pacientes passaram a usar a terapia recomendada pelas diretrizes nos dois períodos analisados. Resultado semelhante foi encontrado em estudo multicêntrico no Canadá, em que 77,0% dos pacientes que não receberam tratamento adequado na alta hospitalar permaneceram sem tratamento adequado após um ano.^3^

Embora não haja estudos nacionais para comparação dos resultados, as prevalências encontradas estão próximas de estudos realizados em outros países.

O estudo apresentou obstáculos que parecem existir entre as evidências e a efetividade desses tratamentos na prática clínica. Mostrou a necessidade do desenvolvimento de estratégias de intervenção para melhoria da qualidade da atenção dada aos pacientes e que se estendam além da alta hospitalar, ponto crítico de transição no cuidado.
